# Maternal circadian disruption during pregnancy and offspring diabetic cardiovascular disease risk

**DOI:** 10.3389/fcell.2026.1879135

**Published:** 2026-08-20

**Authors:** Saman Saedi, Hailong Bing, Jiaxing Wu, Yaoxin Gao, Chunyan Wang, Qinjun Chu, Zhengyuan Xia

**Affiliations:** 1 Department of Anesthesiology and Perioperative Medicine, Zhengzhou Central Hospital Affiliated to Zhengzhou University, Zhengzhou, China; 2 Cell Research and Transformation Center, Zhengzhou Central Hospital Affiliated to Zhengzhou University, Zhengzhou, China; 3 Institute of Trauma and Metabolism, Tianjian Laboratory of Advanced Biomedical Sciences, Academy of Medical Sciences, Zhengzhou University, Zhengzhou, China

**Keywords:** cardiovascular disease, developmental origins of health and disease (DOHaD), diabetic complications, maternal circadian disruption, offspring, oxidative stress

## Abstract

Circadian rhythm disruption during pregnancy has been recognized as a growing public health concern, affecting approximately 20% of the workforce due to shift work and prevalent lifestyle-related factors. Grounded in the developmental origins of health and disease (DOHaD) paradigm, emerging evidence indicates that maternal circadian disruption (MCD) may have profound transgenerational effects, programming offspring susceptibility to diabetes mellitus and its cardiovascular complications. This narrative review synthesizes current mechanistic evidence linking MCD to offspring cardiometabolic disease (CMD), emphasizing the integrated clock-metabolic-oxidative stress axis. Disruption of core clock genes, including *CLOCK*, *BMAL1*, and *CRY*/*PER*, lead to impaired glucose homeostasis, mitochondrial function, and antioxidant defense, subsequently promoting generation of reactive oxygen species (ROS) and exacerbating oxidative stress. These disturbances contribute to endothelial dysfunction, vascular inflammation, and accelerated atherogenesis in offspring. Chronotherapeutic approaches, particularly melatonin supplementation and circadian-aligned lifestyle modifications, represent promising strategies in mitigating these programmed deficits. Elucidating this clock-metabolic-oxidative stress axis will provide essential insights to develop preventive and therapeutic interventions to address the developmental origins of CMD across generations.

## 1 Introduction

Circadian rhythms govern vital physiological functions over a 24-h cycle, coordinating cardiometabolic health parameters such as metabolic homeostasis, blood pressure, and cardiac performance, among other functions (Crowthers et al., 2024; Huart et al., 2023). The spectral composition of ambient light emitted by these sources can be sensed by the skin or the retina, triggering regulation of the endocrine or autonomic nervous system (ANS), which subsequently affects the internal clock (Buhr et al., 2019; Vandewalle et al., 2011). Disruptions to these biological rhythms, caused by factors like shift work, exposure to artificial light at night, and irregular sleep-wake patterns, have emerged as independent risk factors for cardiometabolic health indicators such as metabolic dysfunction, obesity, type 2 diabetes (T2D), hypertension, and cardiovascular disease (CVD) (Knutson et al., 2025). Recent meta-analysis studies demonstrate that shift workers experiencing the brightest nighttime light exposure face 20%–30% increased risks for heart attack, with 26% and 20% higher morbidity risk for coronary heart disease and CVD, respectively, every additional 5 years of exposure (Torquati et al., 2018). Importantly, recent findings have highlighted the consequences of circadian disruption during pregnancy or lactation, propagating to offspring via epigenetic modifications like DNA methylation (Clarkson-Townsend et al., 2019; Marques et al., 2025; Sati et al., 2022).

CVD remains the leading cause of death worldwide, with diabetes significantly contributing to increased rates of cardiovascular morbidity and mortality (Han et al., 2024). The intersection of circadian disruption with diabetes and CVD is particularly concerning given the rising global prevalence of these conditions. A growing body of research suggests that gestational chronodisruption leads to long-term adverse effects on the glucose homeostasis of offspring, the physiology of adipose tissue, and an increased susceptibility to cardiometabolic challenges in adulthood (Cano et al., 2025; Wu et al., 2024). Oxidative stress has recognized as a key pathophysiological mechanism linking diabetes to cardiovascular complications, characterized by an imbalance between the reactive oxygen species (ROS) production and antioxidant defenses (Osman et al., 2025). In the context of diabetes, hyperglycemia exacerbates oxidative stress through various mechanisms, including formation of advanced glycation end products (AGEs), mitochondrial dysfunction, and protein kinase C activation, all of which contribute to increased ROS generation and result in endothelial dysfunction, vascular inflammation, and cardiovascular damage (Chen X. et al., 2025; Nuñez-Selles et al., 2025).

The developmental origins of health and disease (DOHaD) hypothesis posits that environmental exposures during critical developmental stages, especially the prenatal and early postnatal periods, can permanently program offspring physiology and their susceptibility to diseases (Poston et al., 2022). This paradigm has transformed our understanding of the origins of disease and established that early life experiences dramatically affect the risk of chronic diseases in adulthood, which carries considerable implications for public health policy globally (Heindel et al., 2015). Environmental factors during pregnancy, such as maternal nutrition, metabolic status, and stress, have been extensively documented as developmental programming agents that determine health outcomes for offspring across the lifespan (Mirzakhani, 2024). Nevertheless, a less recognized maternal exposure is circadian rhythm disruption, which represents an increasingly prevalent environmental challenge in modern society (Thakur and Kishore, 2025). Recent studies have shown that MCD during pregnancy diminishes placental and neonatal weight, while exacerbating diet-induced obesity in offspring, accompanied by arrhythmic feeding behavior, hypothalamic leptin resistance, and hepatic circadian reprogramming (Yao et al., 2025). Animal studies demonstrate that offspring from mothers exposed to chronic photoperiod shifts suffer alterations in the liver and renal function maturation consistent with adult hypertension, T2D, and obesity (Carmona et al., 2019; Mendez et al., 2019). Additionally, circadian disruption in pregnant women has been associated to heightened risk of pregnancy complications, including hypertension and diabetes, which further compound cardiovascular risks for both mother and offspring that persist into adulthood (Flores et al., 2025).

While understanding these mechanisms holds promise for targeted interventions, direct evidence in offspring remains limited. This narrative review synthesizes the current literature on MCD and its transgenerational cardiometabolic consequences, drawing on mechanistic, epidemiological, and preclinical studies. By elucidating these mechanisms, we aim to provide insights for preventive strategies that address the developmental origins of cardiometabolic diseases (CMD) and to highlight emerging therapeutic possibilities, such as melatonin supplementation and lifestyle modifications that align with circadian biology, ultimately bridging maternal health to cardiovascular outcomes in offspring across generations.

## 2 Core functions and molecular mechanism of circadian rhythms

The mammalian circadian clock is organized hierarchically and regulated centrally in the suprachiasmatic nucleus (SCN) located in the anterior hypothalamus. This nucleus plays a crucial role in coordinating the daily rhythms of peripheral clocks found in various organs throughout the body. The SCN consists of approximately 20,000 neurons organized into distinct ventrolateral and dorsomedial subdivisions, each exhibiting specialized functions in light entrainment and rhythm generation (Abel et al., 2016; Hastings et al., 2018). The ventrolateral SCN receives direct input from the retina via the retinohypothalamic tract, which transmits photic information via glutamate and pituitary adenylate cyclase-activating peptide signaling to adjust the molecular clock in response to environmental light-dark cycles (Colwell, 2011). The facilitation of this photic training is mainly carried out by intrinsically photosensitive retinal ganglion cells that express melanopsin (Berson et al., 2002). The SCN orchestrates peripheral clocks through multiple output pathways, including ANS innervation, endocrine signaling via the hypothalamic-pituitary-adrenal (HPA) axis, and regulation of body temperature rhythms (Kalsbeek et al., 2006). Nearly all peripheral tissues, including liver, heart, kidney, adipose tissue, and skeletal muscle, contain autonomous circadian oscillators that can maintain self-sustained rhythmicity when isolated in culture. However, these peripheral clocks are typically entrained by signals originating from the SCN (Dibner et al., 2010). This hierarchical organization ensures temporal coordination of physiological processes across organ systems, thereby optimizing metabolic efficiency and preventing potentially harmful processes from occurring simultaneously (Bass and Lazar, 2016; Panda, 2016).

**FIGURE 1:**
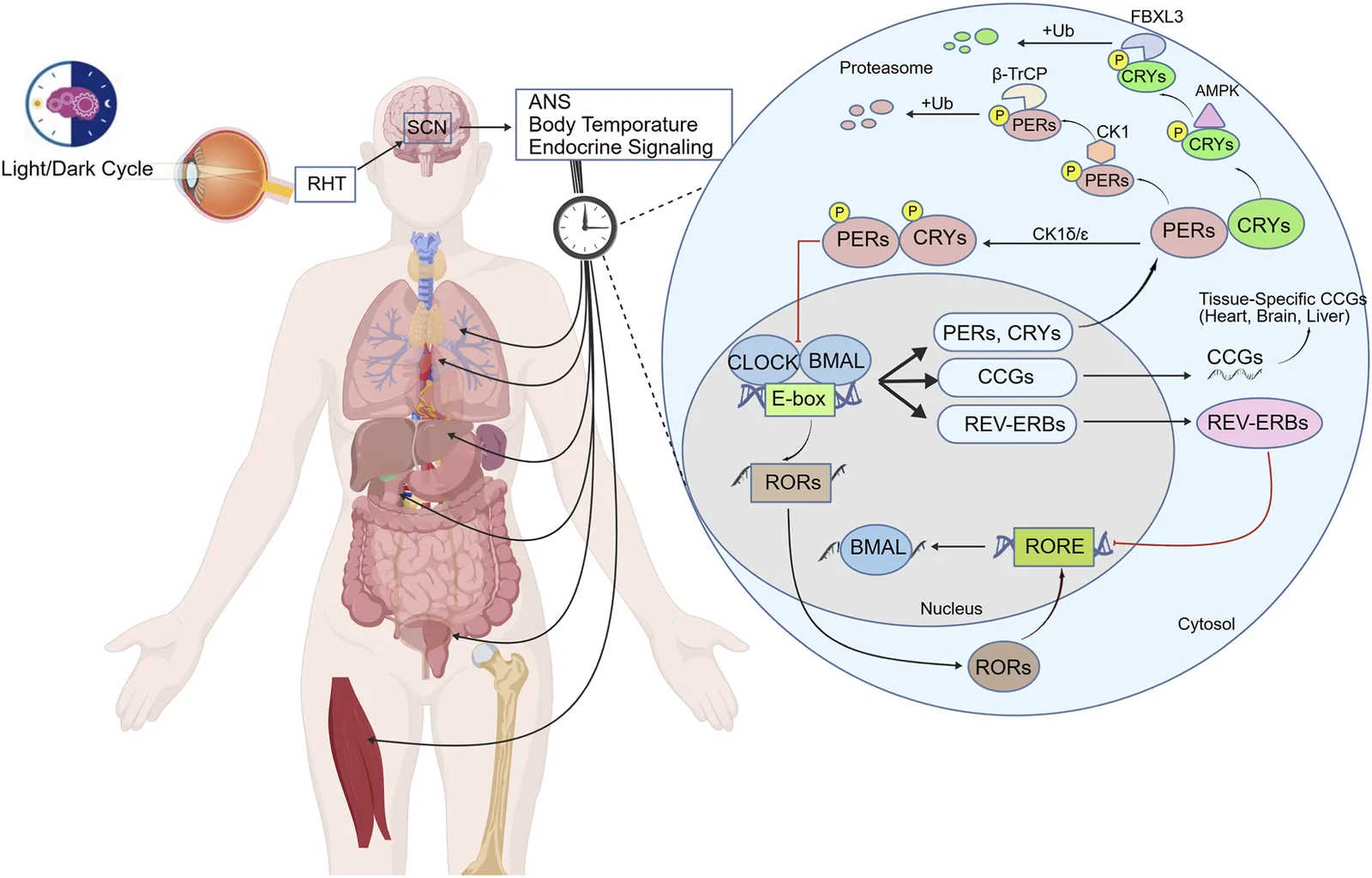
Molecular machinery of the mammalian circadian clock. This schematic illustrates the mammalian circadian clock, integrating central (SCN) and peripheral oscillators, with physiological outputs such as body temperature rhythms. Light entrains the system via RHT to SCN, where CLOCK-BMAL1 activates PER/CRY transcription; stabilized, dephosphorylated PER-CRY complexes accumulate and translocate to the nucleus to repress CLOCK-BMAL1, while CK1δ/ε-mediated phosphorylation promotes PER-CRY degradation and thereby limits this repression. REV-ERBs repress, and RORs activate BMAL1. Figure created in https://biogdp.com. Abbreviations: ANS, autonomic nervous system; BMAL1, brain and muscle ARNT-like 1; CK1δ/ε, casein kinase 1 delta/epsilon; CRY, cryptochrome; PER, period; REV-ERB, reverse Erb; RHT, retinohypothalamic tract; ROR, retinoic acid receptor-related orphan receptor; SCN, suprachiasmatic nucleus.

The molecular machinery underlying circadian rhythms involves an intricate transcription-translation feedback loop that generates oscillations with a period of approximately 24 h (Takahashi, 2017). The positive arm is characterized by the activation of CLOCK (circadian locomotor output cycles kaput) and BMAL1 (brain and muscle Arnt-like protein-1) proteins, which form heterodimers that bind to E-box elements in regulatory regions, activating transcription of period (*PER1-2*) and cryptochrome (*CRY1-2*) genes (Lowrey and Takahashi, 2011). These PER and CRY proteins subsequently heterodimerize in the cytoplasm and translocate to the nucleus to inhibit *CLOCK* and *BMAL1* activity, thereby repressing their own transcription. At a post-transcriptional level, the stability of the PER and CRY proteins is regulated by SCF (Skp1-Cullin-F-box protein) E3 ubiquitin ligase complexes involving β-TrCP and FBXL3, respectively. The kinases, casein kinase 1 delta (CK1δ) and epsilon (CK1ɛ) phosphorylate the PER and CRY proteins to promote polyubiquitination by their respective E3 ubiquitin ligase complexes, which in turn tag the PER and CRY proteins for degradation by the 26S proteasome complex (Mohawk et al., 2012). The balance between these opposing phosphorylation events determines the rate of nuclear accumulation of PER-CRY and, consequently, the period length of the circadian cycle (Reischl and Kramer, 2011; Reischl et al., 2007). The rhythmic expression of *BMAL1* is also modulated by nuclear orphan receptors, including REV-ERBα and REV-ERBβ, as well as retinoic acid receptor-related orphan receptors (RORs), comprising RORα, RORβ, and RORγ. These receptors function to repress and activate the transcription of *BMAL1*, respectively (Meng et al., 2008; Onder and Green, 2018). Overall, this widespread transcriptional regulation is achieved both through direct binding of *CLOCK*-*BMAL1* to E-box elements in gene promoters and through clock-controlled transcription factors that propagate circadian signals to downstream target genes such as *PER1-2*, *CRY1-2*, and *REV-ERBs* (Rey et al., 2011). The major events in the molecular clock of mammals are represented in Figure 1.

## 3 Maternal circadian disruption during pregnancy

### 3.1 Maternal health consequences

Recent evidence suggests that disturbances in maternal circadian rhythms during pregnancy can result in significant consequences for maternal health, extending beyond immediate complications associated with pregnancy to long-term cardiovascular and metabolic risks. Furthermore, studies highlight a link between MCD during pregnancy and an elevated risk of pregnancy complications, especially hypertension and diabetes (Flores et al., 2025). A well-documented outcome of MCD is its association with gestational diabetes mellitus (GDM), which affects approximately 15% of pregnancies worldwide (Lindsay et al., 2017). During pregnancy, disturbances in sleep and circadian rhythms are particularly concerning due to their association with an increased risk of GDM. Women who experience GDM during pregnancy face a significantly greater risk of later developing T2D, CVD, and metabolic syndrome (Mao et al., 2022). The relationship between circadian disruption and GDM appears to be mediated by multiple mechanisms involving glucose homeostasis and metabolic regulation (Meléndez-Fernández et al., 2021). The mechanisms linking circadian disruption to CVD likely involve chronic inflammation, endothelial dysfunction, and ongoing metabolic dysregulation that persists postpartum (Dicom et al., 2023).

Hypertensive disorders of pregnancy, particularly preeclampsia, are also closely linked to circadian disruption, with a distinct and increasingly well-characterized mechanistic basis. Normal pregnancy is characterized by a physiological nocturnal decline (“dipping”) in blood pressure; in preeclampsia, this pattern is frequently replaced by a “non-dipping” or reversed circadian blood pressure profile, which itself predicts adverse maternal and perinatal outcomes independent of absolute blood pressure levels (Salazar et al., 2021; Zhong et al., 2020). At the tissue level, the placenta has a functional molecular clock, and core clock gene transcripts including *BMAL1*, *CLOCK*, *PER1-3*, and *CRY1/2* are co-dysregulated in placentas from women with preeclampsia (Zhou et al., 2022). Placental melatonin synthesis, driven by the enzymes AANAT and ASMT, is also reduced in preeclampsia. Placental macrophages from affected women lose their normal circadian oscillatory capacity even when co-cultured with healthy trophoblasts, suggesting an intrinsic, disease-associated disruption of local clock function rather than a purely secondary phenomenon (Diallo et al., 2023). Consistent with a causal role for circadian disruption, a randomized chronotherapy trial found that shifting low-dose aspirin administration to bedtime, rather than morning, significantly reduced ambulatory blood pressure and the incidence of preeclampsia (Ayala et al., 2013).

Circadian disruption is also mechanistically implicated in intrauterine growth restriction (IUGR), primarily through its effects on placental clock function and uteroplacental perfusion. Clock genes expressed in the placenta regulate angiogenic factors and nutrient-sensing pathways that govern uteroplacental blood flow. Disruption of this placental molecular clock network has been associated with compromised placental function and impaired fetal growth (Bradshaw et al., 2023). In a rat model, gestational immune activation that dysregulated the placental clock gene network simultaneously disrupted maternal blood pressure circadian rhythms and altered fetoplacental growth dynamics, directly linking circadian misalignment to growth-restricted outcomes (Bradshaw et al., 2023). Melatonin, whose rhythmic secretion is itself entrained by the maternal circadian system, further supports fetal growth by enhancing uteroplacental blood flow and mitigating placental oxidative stress. Its supplementation has been proposed as a therapeutic strategy in compromised pregnancies at risk of IUGR (Reiter et al., 2014). However, the mechanisms underlying and the signaling pathways by which MCD increase the risk for T2D, CVD, and metabolic syndrome during pregnancy remain unknown. Further maternal circadian disrupting factors during pregnancy, their mechanisms, and the consequences for maternal health are presented in Table 1.

**TABLE 1:** Maternal circadian disrupting factors during pregnancy, their mechanisms, and the consequences for maternal health.

Factor	Mechanism of circadian disruption	Maternal health consequences	References
Sleep deprivation/Poor sleep quality	Activation of sympathetic nervous system; elevated cortisol; oxidative stress; and altered HPA axis function	Gestational diabetes mellitus, gestational hypertension, preeclampsia, excessive gestational weight gain, and maternal depression	Balserak (2015), Hayase et al. (2014), Okun et al. (2011), Palagini et al. (2016)
Constant light exposure	Complete suppression of melatonin rhythm; disrupted central and peripheral circadian clocks; altered corticosterone profiles; and dysregulation of maternal clock gene expression	Altered gestational length, disrupted metabolic homeostasis, increased inflammation and the risk of chronic disease	Gatford et al. (2019), Mendez et al. (2016), Varcoe et al. (2014)
Chronic phase shifts	Repeated manipulation of light-dark cycle; disruption of maternal circadian rhythms; suppressed melatonin secretion; and peripheral clock gene dysregulation	Disrupted 24-h profiles of activity, altered placental function, and increased maternal stress	Karamitri and Jockers (2019), Varcoe et al. (2011), Wallace et al. (2023)
Night shift work	Suppression of melatonin; circadian misalignment; elevated catecholamines; and increased maternal stress response	Preterm delivery, miscarriage, gestational diabetes, and hypertensive disorders of pregnancy	Cai et al. (2019), Izadi et al. (2024), Kader et al. (2021), Lee et al. (2024)
Artificial light at night (ALAN)	Suppression of melatonin secretion; disruption of circadian clock genes; altered hormonal balance; and phase delay of circadian rhythm	Sleep disorders, increased risk of preterm birth, preeclampsia, chronic placental insufficiency, miscarriage, and gestational hypertension	Sun et al. (2023), Zhang et al. (2024), Zou et al. (2025)
Mistimed eating	Desynchronization between central and peripheral clock and altered metabolic timing	Impaired glucose homeostasis, increased risk of gestational diabetes and metabolic dysfunction	Fishbein et al. (2021), Weschenfelder et al. (2021)

### 3.2 Offspring health consequences

Maternal circadian disruption (MCD) during pregnancy produces offspring effects spanning metabolic, cardiovascular, and even multigenerational domains, with the placenta serving as the principal mediator of maternal-fetal circadian communication. The placenta harbors a functional molecular clock, and disruption of its core clock genes compromises placental nutrient transfer efficiency, providing a unifying mechanistic link between MCD and the diverse offspring phenotypes described below (Mark et al., 2017; Wharfe et al., 2016; Yao et al., 2025). The specific molecular pathways connecting placental clock disruption to metabolic and cardiovascular programming are described in detail in Sections 4 and 5.

The most consistently reported offspring health consequences are metabolic and growth-related. MCD alters the normal phase relationship between mother and fetus, with lasting effects on offspring metabolic health (Cui et al., 2024; Yao et al., 2025), while maternal comorbidities associated with MCD compound this risk: maternal hyperglycemia in GDM crosses the placenta and impairs fetal growth (Flores et al., 2025). Notably, the timing of MCD determines the severity and nature of these outcomes. Yao et al. (2025) demonstrated that circadian disruption during the third trimesters of pregnancy was sufficient to induce offspring phenotypes, including altered growth, impaired glucose tolerance, hepatic steatosis, and elevated systolic blood pressure with vascular stiffness in adult offspring, indicating that late gestation is a critical developmental window for cardiometabolic programming. Importantly, most of this evidence characterizes offspring disease levels such as elevated blood pressure rather than the underlying rhythmicity of physiological function. Direct assessment of offspring circadian physiology remains uncommon, but where it has been examined, it reveals that MCD disrupts not only the magnitude but also the temporal organization of cardiovascular function. In addition, adult rat offspring exposed to maternal chronic photoperiod shifts display altered diurnal rhythms of both blood pressure and heart rate, characterized by elevated nocturnal systolic blood pressure and increased rhythm amplitude relative to controls (Varcoe et al., 2018). Nevertheless, comparable direct measures of offspring rhythmicity, such as actigraphy-derived sleep-wake patterns or ambulatory blood pressure monitoring, remain largely unexplored in human cohorts.

The consequences of MCD affect physiological systems and potentially multiple generations. Animal studies have illustrated that lesioning the maternal SCN or knocking out the maternal core clock genes, *Per1* and *Per2*, prevented entrainment of the fetal circadian system (Jud and Albrecht, 2006; Reppert and Schwartz, 1986). This observation supports the hypothesis that input from the maternal circadian system entrains fetal daily rhythms. Potential maternal signals implicated in fetal circadian synchronization include glucocorticoids, melatonin, and dopamine (Mark et al., 2017). Emerging evidence suggests this disruption can propagate transgenerationally through epigenetic mechanisms: chronic gestational circadian disturbance produces mood disorder-like behaviors persisting through the F1 and F2 generations in a sex-specific manner, accompanied by elevated corticosterone and disrupted clock gene expression, including *Per1*, *Per2*, *Bmal1*, *Clock* in the F1 SCN (Zhang et al., 2017). While altered DNA methyltransferase (DNMT) expression in the testicular germline of exposed offspring provides a plausible mechanism for transmitting circadian disruption to subsequent, unexposed generations (Tas et al., 2022). However, future research should focus on consequences of MCD in offspring, particularly specific epigenetic markers, such as histone modifications, in conjunction with DNMTs and the role of maternal signals in germline transmission. Table 2 summarizes maternal circadian disruptors discussed here alongside their associated offspring health consequences.

**TABLE 2:** Maternal circadian disrupting factors during pregnancy, their mechanisms, and health consequences in offspring.

Factor	Mechanism of circadian disruption	Offspring health consequences	References
Night shift work	Disrupted maternal-fetal circadian synchronization; altered placental function; and reduced maternal melatonin affecting fetal neurodevelopment	Low birth weight, metabolic syndrome risk, cardiovascular health impacts, and potentially altered mental health in young adult offspring	Flores et al. (2025), Li et al. (2023), Strohmaier et al. (2019), Strohmaier et al. (2018)
Constant light exposure	Suppressed maternal melatonin crossing the placenta and activation of renin-angiotensin system	Hypertension in adult offspring and long-term adverse behavioral and biochemical consequences	Landgraf et al. (2014), Stoyanova et al. (2025)
Chronic phase shifts	Disrupted maternal melatonin rhythm affecting fetal tissues; altered glucocorticoid signaling; epigenetic modifications; and disrupted fetal SCN development	Increased adiposity, poor glucose tolerance and metabolic dysfunction, increased anxiety-like behaviors, elevated blood pressure, and altered corticosterone rhythm	Salazar et al. (2018), Varcoe et al. (2013), Yao et al. (2025)
Maternal sleep disruption/deprivation	Disrupted maternal hormone rhythms; impaired placental function; elevated maternal inflammatory cytokines; altered maternal metabolism affecting fetal programming	Reduced hippocampal neurogenesis, brain immaturity at birth, higher offspring BMI, and blood pressure	Hoyniak et al. (2025), Kim et al. (2020), Sanapo et al. (2024), Vanderplow et al. (2022), Zhao et al. (2014)
Artificial light at night (ALAN)	Suppressed maternal melatonin affects fetal circadian development; altered development of fetal; disrupted hormonal rhythmicity in offspring	Altered fetal size and thyroid hormones, disrupted glucose and cholesterol rhythms, altered functions of neurological system, and endocrine disruption	Dzirbíková et al. (2022), WANG et al. (2025), Zhang et al. (2024), Zhang et al. (2023)

## 4 Maternal circadian disruption and offspring diabetes risk

### 4.1 Mechanisms underlying metabolic dysfunction

Although the link between maternal circadian rhythms during pregnancy and offspring diabetes is poorly understood, experimental research indicates that disruptions in maternal circadian rhythms during pregnancy dramatically increase offspring susceptibility to glucose intolerance and diabetes (Flores et al., 2025). Offspring exposed to maternal shift work patterns exhibit increased fasting glucose levels, impaired glucose tolerance, and diminished insulin sensitivity (Halabi et al., 2021; Mendez et al., 2016). Moreover, maternal shift work during pregnancy was associated with an increased risk of insulin resistance in early childhood (Liao et al., 2022). Varcoe et al. (2011) reported that pregnant rats exposed to chronic photoperiod shifts exhibited offspring with impaired glucose tolerance, hyperinsulinemia, and a decrease in pancreatic β-cell mass in adulthood. These metabolic alterations were linked to altered expression of essential clock genes, including *Bmal1* and *Clock*, in the pancreatic tissues of the offspring (Varcoe et al., 2011). The SCN, which serves as the primary circadian pacemaker, regulates peripheral clocks in metabolic tissues, including liver, pancreas, and adipose tissue through neural and humoral signals (Dibner et al., 2010; Kolbe, 2017). MCD leads to the desynchronization of these peripheral clocks in the developing fetus, establishing discordant metabolic rhythms that persist postnatally (Matsumura et al., 2016). Gestational chronodisruption may act through epigenetic mechanisms to define an abnormal adipose tissue phenotype, which is functionally consistent with increased risk of obesity, insulin resistance, and diabetes (Halabi et al., 2021). Moreover, Phase-shifted expression of these clock genes in offspring of circadian-disrupted mothers establishes a state of chronically dysregulated hepatic glucose production and reduced insulin sensitivity, creating a developmental substrate for later-onset glucose intolerance and T2D (Flores et al., 2025). These effects can be caused through the clock components (*CLOCK* and *BMAL1*) or downstream target genes, such as *PER1-2*, *CRY1-2*, and *REV-ERBs*. Pancreatic β-cell development and function are significant targets affected by MCD. Disruption of the clock components *CLOCK* and *BMAL1* leads to hypoinsulinemia and diabetes through widespread alterations in pancreatic β-cell gene expression (Marcheva et al., 2010). However, the precise roles of clock gene components and their downstream targets in regulating insulin secretion and glucose metabolism remain unclear in offspring of mothers exposed to circadian disruption.

**FIGURE 2:**
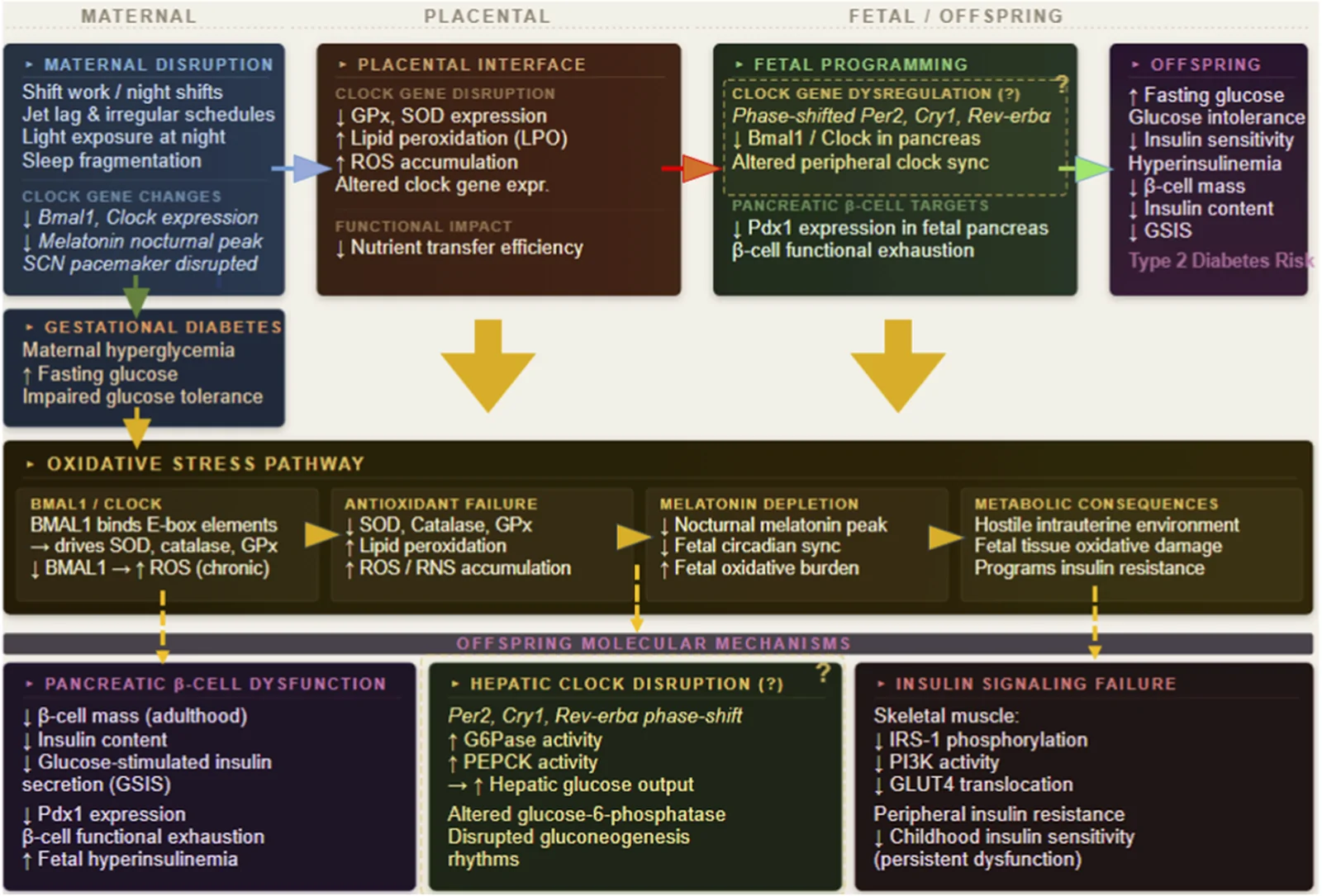
Mechanistic pathways linking maternal circadian rhythm disruption to offspring diabetes risk. The diagram illustrates three interconnected axes: (i) the metabolic dysfunction pathway, in which shift work and exposure to light-at-night disrupt the maternal SCN, leading to changes in peripheral clock gene expression (*Bmal1*, *Clock*) and an increased risk of gestational diabetes; (ii) the placental oxidative interface, where circadian disruption diminishes the expression of placental GPx, catalase, and SOD, resulting in elevated levels of ROS and LPO; and (iii) the fetal programming layer, in which probably phase-shifted clock genes (*Per2*, *Cry1*, *Rev-erbα*) and reduced Pdx1 expression impair β-cell development and function. The oxidative stress pathway (amber) acts bidirectionally, exacerbated by blunted melatonin signaling and *BMAL1*-driven loss of antioxidant rhythm. These converging mechanisms lead to impaired insulin secretion, excessive hepatic glucose production, and peripheral insulin resistance in postnatal offspring. Hypothetical and poorly characterized pathways are shown with amber dashed lines to distinguish them from well-established mechanisms. GSIS, glucose-stimulated insulin secretion; G6Pase, glucose-6-phosphatase; PEPCK, phosphoenolpyruvate carboxykinase; IRS-1, insulin receptor substrate-1; PI3K, phosphatidylinositol 3-kinase; Pdx1, pancreatic and duodenal homeobox 1; ROS, reactive oxygen species; SCN, suprachiasmatic nucleus.

As mentioned before, during pregnancy, sleep and circadian disturbances are particularly concerned due to their association with maternal hyperglycemia and a heightened risk of GDM (Mao et al., 2022; Meléndez-Fernández et al., 2021). Fetal hyperinsulinemia, resulting from maternal hyperglycemia, programs pancreatic β-cell dysfunction and peripheral insulin resistance in offspring. β-cells exposed to elevated glucose during development undergo functional exhaustion, with reduced insulin secretory capacity in postnatal life (Aerts and Van Assche, 2006). Besides, studies indicate that offspring of circadian-disrupted mothers have reduced β-cell mass, decreased insulin content, and impaired glucose-stimulated insulin secretion (Qian et al., 2013). The underlying molecular mechanisms involve disrupted expression of pancreatic and duodenal homeobox 1, which is a key regulator of β-cell development and function, in fetal pancreatic tissue (Rakshit et al., 2014). Additionally, insulin signaling pathways in peripheral tissues demonstrate persistent dysfunction in offspring of circadian-disrupted mothers. Skeletal muscle from these offspring exhibits diminished insulin receptor substrate-1 (IRS-1) phosphorylation, decreased phosphatidylinositol 3-kinase (PI3K) activity, and impaired GLUT4 translocation in response to insulin stimulation (Niu et al., 2015). The mechanisms linking MCD to offspring diabetes risk are shown in Figure 2.

### 4.2 Mechanisms underlying oxidative stress and circadian dysfunction

The bidirectional relationship between circadian rhythms and redox homeostasis during pregnancy influences long-term offspring metabolic health. Oxidative stress arises from an imbalance between ROS/RNS production and antioxidant defenses such as SOD, catalase, and GPx (Tain and Hsu, 2024). During pregnancy, physiological ROS supports oocyte maturation (Guerin et al., 2001), placental (Myatt, 2010), and fetal development (Tain and Hsu, 2024), but MCD tips this balance toward excess oxidative stress, programming offspring susceptibility to diabetes.

During pregnancy, MCD amplifies oxidative stress in the placenta, a critical interface between maternal and fetal environments (Zhang et al., 2025). This placental oxidative stress compromises nutrient transfer efficiency and alters the expression of placental clock genes, creating a hostile intrauterine environment that programs fetal metabolic tissues toward insulin resistance (Astiz et al., 2020). At the molecular level, core clock genes including *BMAL1*, *CLOCK*, *PER*, and *CRY* orchestrate rhythmic expression of antioxidant defense enzymes, establishing temporal coordination of cellular redox capacity (Rv, 2006). *BMAL1*, which is the positive component of the circadian clock, directly binds to E-box regulatory elements in the promoter regions of genes encoding SOD, catalase, and peroxisomal enzymes, driving their circadian oscillation (Pekovic-Vaughan et al., 2014). Disruption of *BMAL1* expression results in constitutively elevated ROS levels and accelerated accumulation of oxidative damage in multiple tissues (Kondratova and Kondratov, 2012).

Additionally, maternal melatonin, which normally synchronizes fetal circadian rhythms and provides antioxidant protection, exhibits diminished nocturnal peaks in the presence of circadian disruption, thereby increasing the oxidative burden on the fetus (Figure 2) (Mendez et al., 2016). Indeed, melatonin is not only a circadian hormone but also a potent antioxidant that acts through both direct and indirect mechanisms. Directly, melatonin scavenges ROS/RNS, such as hydroxyl radical (⋅OH), hydrogen peroxide, and peroxynitrite, partly through its indole ring and a resonance-stabilized electron-donating capacity (Chrustek and Olszewska-Słonina, 2021). Melatonin downstream metabolites, such as AFMK and AMK, also retain antioxidant activity, thereby contributing to a melatonin antioxidant cascade. Indirectly, melatonin acts via melatonin receptors (MT)1 and 2 signaling to upregulate the expression and activity of antioxidant enzymes, including SOD, GPx, and catalase, thereby strengthening cellular redox defense (Galano and Reiter, 2018). In the placenta, where melatonin is locally synthesized, reduced melatonin levels have been associated with placental oxidative stress, preeclampsia, and intrauterine growth restriction, supporting a link between circadian disruption, impaired antioxidant defense, and adverse pregnancy outcomes (Aversa et al., 2012). These functions of melatonin are discussed in more detail in Section 6. In summary, these results suggest that oxidative stress represents both a consequence and mediator of metabolic dysfunction, particularly diabetes in offspring subjected to MCD. Circadian rhythms directly regulate antioxidant defense systems, and the disruption of clock gene expression can lead to significant consequences. As will be discussed in Section 5.1, this same placental oxidative substrate that programming offspring diabetes risk also underlies subsequent cardiovascular complications.

## 5 Maternal circadian disruption and offspring diabetic cardiovascular complications

### 5.1 Shared oxidative substrate linking offspring diabetes to cardiovascular risk

**FIGURE 3:**
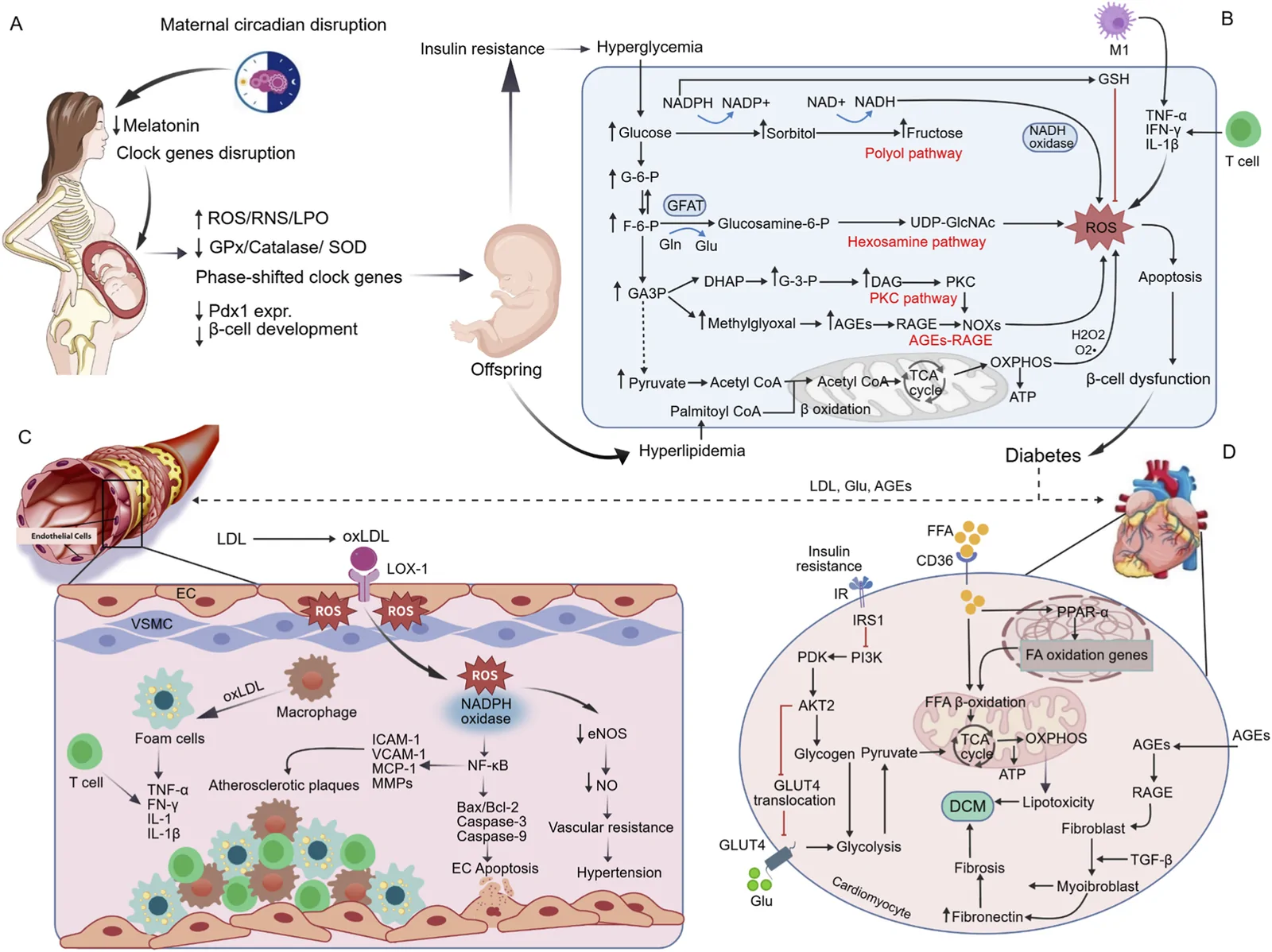
Mechanistic pathways linking MCD and offspring diabetic cardiovascular complications. **(A)** MCD reduces melatonin secretion, dysregulates clock genes, elevates ROS/RNS/LPO, impairs antioxidant defenses (GPx, catalase, SOD), and suppresses Pdx1-mediated β-cell development, collectively inducing insulin resistance and fetal hyperglycemia. **(B)** Chronic hyperglycemia activates four intracellular pathways, including polyol, hexosamine (GFAT), PKC, and AGEs–RAGE. It converges on overproduction of NADPH oxidase-derived ROS and mitochondrial OXPHOS dysregulation, leading to β-cell apoptosis and dysfunction in offspring. **(C)** Diabetes- and hyperlipidemia-driven elevation of LDL, glucose, and AGEs promotes endothelial uptake of oxLDL via LOX-1. oxLDL uptaking triggers NADPH oxidase-mediated ROS generation, eNOS/NO suppression, NF-κB activation, and upregulation of ICAM-1, VCAM-1, MCP-1, and MMPs, facilitating monocyte infiltration, foam cell formation, atherosclerotic plaque progression, endothelial apoptosis (caspase-3/9), and hypertension. **(D)** In cardiomyocytes, impaired IR/IRS1/PI3K/AKT2 signaling reduces GLUT4-mediated glucose uptake; compensatory CD36-driven FFA uptake activates PPARα-dependent β-oxidation, causing lipotoxicity. Simultaneously, AGEs–RAGE and TGF-β induce cardiac fibroblast-to-myofibroblast transition, leading to interstitial fibrosis, culminating in DCM. Hypothetical pathways linking MCD and offspring diabetic cardiovascular complications are shown with dashed lines. Figure created in https://biogdp.com. Abbreviations: MCD, maternal circadian disruption; AGEs, advanced glycation end-products; AKT2, protein kinase B isoform 2; catalase, hydrogen peroxide–decomposing antioxidant enzyme; CD36, fatty acid translocase; DAG, diacylglycerol; DCM, diabetic cardiomyopathy; eNOS, endothelial nitric oxide synthase; FFA, free fatty acid; GFAT, glutamine-fructose-6-phosphate aminotransferase; GLUT4, glucose transporter type 4; GPx, glutathione peroxidase; ICAM-1, intercellular adhesion molecule 1; IFN-γ, interferon-gamma; IL-1β, interleukin-1 beta; IR, insulin receptor; IRS1, insulin receptor substrate 1; LDL, low-density lipoprotein; LOX-1, lectin-like oxidized LDL receptor-1; LPO, lipid peroxidation; MCP-1, monocyte chemoattractant protein 1; MMPs, matrix metalloproteinases; NADPH oxidase, nicotinamide adenine dinucleotide phosphate oxidase; NF-κB, nuclear factor kappa-light-chain-enhancer of activated B cells; NO, nitric oxide; OXPHOS, oxidative phosphorylation; oxLDL, oxidized low-density lipoprotein; Pdx1, pancreatic and duodenal homeobox 1; PI3K, phosphoinositide 3-kinase; PKC, protein kinase C; PPAR-α, peroxisome proliferator-activated receptor alpha; RAGE, receptor for advanced glycation end-products; RNS, reactive nitrogen species; ROS, reactive oxygen species; SOD, superoxide dismutase; TGF-β, transforming growth factor beta; TNF-α, tumor necrosis factor alpha; VCAM-1, vascular cell adhesion molecule 1.

Although previous studies have shown an association between circadian disruption and cardiovascular health, few have specifically investigated diabetic cardiovascular complications in offspring exposed to MCD during pregnancy. As discussed in Section 4, MCD-driven placental oxidative stress and impaired β-cell programming establish a hyperglycemic, insulin-resistant metabolic milieu in offspring (Yao et al., 2025). In the insulin resistance condition, the cells in the muscles, adipose, and liver fail to respond adequately to insulin, resulting in sustained hyperglycemia. This hyperglycemia drives ROS overproduction through elevated mitochondrial respiration and protein kinase C (PKC)-dependent nicotinamide adenine dinucleotide phosphate (NADPH) oxidase activity, while enhancing pro-oxidative flux through the AGE, hexosamine, and polyol pathways (Osman et al., 2025; Papachristoforou et al., 2020). Polyol pathway activation additionally depletes NADPH and impairs glutathione (GSH) regeneration, further reducing cellular antioxidant capacity (Xie et al., 2020). These major molecular pathways are remarkable risk pathways for apoptosis in β-cells, resulting diabetes. Indeed, prolonged oxidative stress promotes the development of diabetes and its complications by impairing the insulin secretion of β-cell, raising insulin resistance, and inducing systemic inflammation (Osman et al., 2025). Prolonged oxidative stress of this kind promotes β-cell apoptosis and contributes to both the microvascular (retinopathy, nephropathy, neuropathy) and macrovascular (cardiovascular, cerebrovascular, peripheral arterial) complications of diabetes (Papachristoforou et al., 2020; Zhang et al., 2020). MCD exacerbates oxidative stress in the placenta during pregnancy (Zou et al., 2025), which dwindles placental expression of GPx and SOD, resulting in the accumulation of ROS and lipid peroxidation (LPO) products (Figure 3A) (Asik et al., 2024). The excessive ROS in β-cells due to hyperglycemia and hyperlipidemia generates ox-insulin peptides. It is subsequently stimulates the reactivity of proinflammatory T lymphocytes (T cell) specifically against β-cells (Chen X. et al., 2025). Besides, M1-type macrophages in pancreatic islets generate and release inflammatory cytokines like interferon-gamma (IFN-γ), interleukin (IL)-1β and tumor necrosis factor-alpha (TNF-α) due to intracellular ROS, causing β-cells apoptosis (Figure 3B) (Chen X. et al., 2025). Together, these metabolic and immune-mediated pathways establish the oxidative and inflammatory substrate that links offspring diabetes risk to the coronary and myocardial complications examined in Sections 5.2, 5.3.

### 5.2 Maternal circadian disruption-induced diabetes may contribute to coronary artery disease risk in offspring

Coronary artery disease (CAD) has emerged as a very common cardiovascular complication of diabetes mellitus, driven by a complex interplay of metabolic dysregulation, chronic inflammation, and progressive oxidative injury to the vascular endothelium (Jia et al., 2018; Kenny and Abel, 2019). LPO is a pathogen-associated molecular pattern in the immune system, which triggers chronic inflammation and the development of diabetic cardiovascular complications (Binder et al., 2016). In the early stages of atherosclerosis, a major cause of CAD, myocardial infarction, and stroke, ROS generated through the enzymatic activity of lysyl oxidase and myeloperoxidase in the artery wall aggravated the oxidation and glycosylation of low-density lipoproteins (LDLs), contributing to elevated circulating levels of oxidized LDLs (oxLDLs) (Jiang et al., 2022). These oxLDLs are recognized and internalized primarily through the lectin-like oxidized LDL receptor-1 (LOX-1), a scavenger receptor constitutively expressed on the surface of vascular endothelial cells (ECs) and vascular smooth muscle cells (VSMCs) (Pirillo et al., 2013). Through LOX-1, oxLDLs exert direct cytotoxicity on ECs and simultaneously act as potent chemoattractants for circulating immune cells, including macrophages and T cells, which adhere to and infiltrate the activated (Balci et al., 2024; Tang et al., 2022). These immune cells release inflammatory cytokines such as TNF-α, IFN-γ, IL-1, and IL-1β, which further encourage the chemotaxis of these immune cells toward developing atherosclerotic plaques (Herrero-Fernandez et al., 2019). On the other hand, internalization of oxLDLs drives the differentiation of macrophages into foam cells, which collaborate with macrophages to exacerbate the inflammatory effect and oxidative damage (Figure 3C) (Marchio et al., 2019). The interplay of oxLDLs, endothelial cells, and immune cells promotes atherosclerosis (Liu et al., 2020).

In offspring of circadian-disrupted mothers, the resulting diabetic milieu enriched with AGEs, ox-LDL, free fatty acids (FFA), and hyperglycemia-derived glucose, LOX-1 engagement triggers marked upregulation of ROS, ⋅OH generation, NADPH oxidase activation, and uncoupling of endothelial nitric oxide synthase (eNOS) (Barreto et al., 2021; Pirillo et al., 2013). These oxLDLs are taken up via the LOX-1 scavenger receptor on vascular endothelial cells, triggering NADPH oxidase-mediated ROS generation, activation of nuclear factor kappa-B (NF-κB), upregulation of ICAM-1, VCAM-1, and MCP-1, monocyte infiltration, and foam cell formation. Collectively driving atherosclerotic plaque progression and CAD in offspring (Hu et al., 2025; Stancel et al., 2016). Meanwhile, diabetes-driven ROS/RNS further impair vascular homeostasis by suppressing nitric oxide synthase (eNOS)-derived nitric oxide (NO) bioavailability, through PKC-mediated eNOS inhibition and superoxide-driven conversion of NO to peroxynitrite. This precipitates a self-reinforcing cycle of NO deficiency, platelet aggregation, VSMC proliferation, and chronic hypertension (Figure 3C) (Kumar et al., 2019; Meza et al., 2019).

### 5.3 Maternal circadian disruption-induced diabetes and cardiomyopathy in offspring

Diabetic cardiomyopathy (DCM) is a distinct form of cardiac dysfunction that develops in patients with T2D independently of coronary artery disease or hypertension. Leptin resistance contributes to pathogenesis of DCM through impaired cardiac glucose uptake and increased reliance on fatty acid (FA) oxidation with associated lipotoxicity (Boudina and Abel, 2007). In the DCM, chronically elevated circulation and local leptin levels paradoxically fail to activate these protective cascades. The concept of “cardiac leptin resistance” has emerged, wherein elevated local and systemic leptin levels fail to activate protective signaling pathways in cardiomyocytes while promoting hypertrophic and fibrotic responses (Karmazyn et al., 2007). At the metabolic level, impaired leptin signaling in cardiomyocytes shifts substrate utilization away from glucose toward excessive FA oxidation. This increased dependence on FA oxidation generates ROS, and promotes lipid intermediary accumulation, collectively termed lipotoxicity, which impairs mitochondrial function and contractile performance. In DCM, insulin-dependent glucose uptake signaling is markedly diminished, leading to compensatory upregulation of FA uptake via the CD36 transporter and eventually lipid accumulation within cardiomyocytes. Under normal physiological conditions, insulin activates the IR/IRS-1/PI3K/AKT2 axis in cardiomyocytes, promoting GLUT4 membrane translocation and glucose oxidation via glycolysis and the TCA cycle. The consequent increase in FA uptake and PPAR-α–driven FA oxidation gene expression is initially adaptive but rapidly becomes maladaptive: the mitochondria are unable to efficiently oxidize the excessive FA flux, resulting in accumulation of lipid intermediates including ceramide and diacylglycerol, generation of ROS, and mitochondrial uncoupling, a constellation termed lipotoxicity. The excessive FA load exceeds the mitochondrial respiratory capacity to generate ATP, ultimately leading to cardiomyocyte death, impaired contractile function, and progressive lipid accumulation. On the other hand, AGEs bind to their receptor RAGE and stimulate transforming growth factor-β secretion from activated inflammatory cells. This cytokine subsequently induces cardiac fibroblast-to-myofibroblast transition, enhances fibronectin and collagen deposition, and inhibits matrix metalloproteinase activity via TIMP overexpression, leading to interstitial fibrosis and increased myocardial stiffness. Together, these interlocking mechanisms, metabolic inflexibility, lipotoxicity, ROS overproduction, and fibrotic remodelling, culminate in the clinical phenotype of DCM: diastolic dysfunction progressing to systolic failure and, ultimately, overt CAD (Figure 3D) (Tan et al., 2020). However, human evidence remains limited to associative cohorts versus causal animal data. Direct evidence of MCD-specific CAD/DCM in human offspring is lacking.

## 6 Therapeutic considerations and preventive strategy

Evidence-based treatment and preventive approaches must be developed in light of the growing awareness that maternal circadian disturbance during pregnancy promotes the risk of diabetic cardiovascular complications in offspring. The long-term transgenerational effects as well as the current maternal health issues must be addressed by these approaches. Nevertheless, current methods concentrate on two complementary strategies: pharmaceutical therapies based on chronotherapy (melatonin) and chrononutrition (melatonin precursors), and extensive lifestyle alterations in line with circadian principles. In order to maximize maternal-fetal outcomes and avoid the developmental programming of diabetic CMD, these strategies must be implemented with careful consideration of timing, dosage, safety profiles, and individual patient features.

### 6.1 Melatonin and chronotherapy

The multifaceted therapeutic potential of melatonin in pregnancy stems from its integrated actions on circadian synchronization, antioxidant defense, and metabolic regulation. By activating MT1/MT2, which are found in metabolically active tissues such as the liver, pancreas, adipose tissue, and cardiovascular system, melatonin mechanistically synchronizes peripheral circadian clocks (Karamitri and Jockers, 2019). Timed maternal melatonin therapy efficiently restores normal circadian rhythmicity in offspring by reversing the circadian disturbance of the fetal adrenal clock caused by continuous light exposure, as shown in animal experiments (Mendez et al., 2012). Additionally, melatonin acts as a potent direct and indirect antioxidant, scavenging ROS and upregulating GPx, SOD, and catalase activity during pregnancy (Tain and Hsu, 2024). A recent animal study has demonstrated that melatonin supplementation prevented the chronic photoperiod shifting during pregnancy, abnormal pro-inflammatory cytokine patterns such as IL1a and IL6, and cardiovascular and steroidogenic gene expression alteration (Mendez et al., 2022), supporting its potential as a protective intervention against pregnancy complications driven by oxidative stress.

Chronotherapy is an emerging approach that extends beyond melatonin supplementing. It involves strategically scheduling medication administration and therapeutic interventions in accordance with circadian rhythms to enhance effectiveness and reduce adverse effects. In cardiovascular medicine, chronotherapeutic strategies have shown promise in improving treatment outcomes by aligning drug delivery with endogenous circadian variations in physiological processes (Khoshnevis et al., 2024). In the context of diabetes and MCD, chronotherapy could be utilized to optimize the timing of insulin administration and glucose metabolism though this application requires rigorous investigation through well-designed clinical trials (Singh et al., 2009).

Overall, clinical translation of these approaches requires attention to intervention timing; the third trimester is a particularly sensitive window for fetal programming (Yao et al., 2025). Nevertheless, individualization should be based on chronotype, shift-work status, and cardiometabolic risk. Future large-scale randomized controlled trials should evaluate melatonin’s safety and efficacy using standardized dosing and circadian-phase-timed administration, incorporating both maternal circadian biomarkers such as dim-light melatonin onset and actigraphy, and offspring cardiometabolic outcomes extending into childhood.

### 6.2 Chrononutrition: tryptophan and serotonin

Beyond exogenous melatonin administration, nutritional strategies targeting the tryptophan–serotonin–melatonin biosynthetic pathway represent a complementary, and arguably more accessible, chrononutritional approach. Tryptophan is the essential amino acid precursor of serotonin, which is in turn enzymatically converted to melatonin via serotonin N-acetyltransferase and acetylserotonin O-methyltransferase (Xie et al., 2022). Its dietary availability during pregnancy is influenced by maternal diet, gut microbial metabolism, and placental transport (Badawy, 2015). Notably, the evidence supporting tryptophan-based chrononutrition is considerably stronger in the offspring than in the pregnant mother herself. In human infants, tryptophan-enriched formula milk and breast milk with a preserved circadian tryptophan rhythm have been shown to consolidate the infant sleep–wake cycle and increase nocturnal melatonin and serotonin metabolite levels (Cubero et al., 2009; Sanchez, 2005). In an animal model, maternal tryptophan supplementation during pregnancy protected adult offspring against hypertension programmed by maternal chronic kidney disease, an effect linked to modulation of the tryptophan-metabolizing gut microbiome and aryl hydrocarbon receptor signaling (Hsu et al., 2020). By contrast, to our knowledge, no human trials have directly evaluated maternal tryptophan, serotonin, or other melatonin-precursor supplementation during pregnancy for the specific purpose of restoring maternal circadian rhythmicity or preventing MCD-associated gestational or cardiovascular outcomes. This represents an important gap, given that such nutritional interventions may carry a more established safety profile in pregnancy than pharmacological melatonin and could, in principle, be combined with the chronotherapeutic strategies discussed below.

### 6.3 Lifestyle interventions

Lifestyle interventions targeting circadian health represent practical, safe, and potentially effective approaches to prevent MCD and its transgenerational consequences. These approaches focus on enhancing the key environmental and behavioral synchronizers of circadian rhythms like light exposure, sleep timing and quality, physical activity patterns, and meal timing. The American Heart Association’s 2025 Scientific Statement on Circadian Health highlights that interventions aimed at these synchronizers can improve circadian health and reduce circadian disruptions, especially concerning the prevention of CMD (Knutson et al., 2025). Notably, lifestyle interventions during pregnancy provide the dual advantage of promoting maternal health and affecting offspring developmental programming while avoiding potential issues associated with pharmacological treatments.

Sleep hygiene recommendations particularly relevant for pregnant women include maintaining consistent sleep-wake schedules even on weekends, creating a bedroom environment conducive to sleep characterized by a cool temperature, minimal noise and light, and comfortable bedding, avoiding caffeine after early afternoon, limiting fluid intake before bedtime to decrease nocturia, and establishing a relaxing pre-sleep routine (Chen P. et al., 2025). Social jetlag, which refers to the difference in sleep timing between workdays and days off, is a significant yet often overlooked factor that contributes to circadian misalignment. Reducing social jetlag by adhering to regular sleep schedules throughout the week helps maintain circadian stability and improves metabolic health (Knutson et al., 2025). For shift-working pregnant women, strategic napping protocols, optimized shift rotation schedules, and making workplace adjustments to lessen the frequency of night shifts during pregnancy may assist in mitigating the impacts of circadian disruption. However, additional research is required to establish evidence-based guidelines for this at-risk population.

## 7 Conclusion

MCD during pregnancy programs offspring cardiometabolic risk primarily through a disrupted clock–metabolic–oxidative stress axis, leading to impaired glucose homeostasis, increased oxidative damage, and vascular dysfunction. These mechanistic links suggest that maternal chronodisruption contributes to offspring susceptibility to diabetes and its cardiovascular complications, including atherosclerosis and diabetic cardiomyopathy. Preventive strategies that restore maternal circadian alignment, such as timed light exposure, sleep hygiene, and targeted melatonin supplementation, alongside further mechanistic and longitudinal human studies, are warranted. Focusing future research on placental clock function, epigenetic transmission, and translational chronotherapeutics could identify actionable interventions to reduce transgenerational cardiometabolic disease burden.

### 7.1 Limitation

Despite mechanistic insights from animal models linking MCD to diabetic CVD in offspring, several limitations persist: most evidence derives from rodent studies using artificial chronodisruption with limited human interventional trials to establish causality. Observational cohorts of shift workers show associations with gestational diabetes mellitus and cardiometabolic risk in offspring; however, long-term cardiovascular endpoints such as myocardial infarction and stroke remain untracked. MCD often coexists with confounders such as obesity, poor diet, and socioeconomic factors, which complicates the isolation of independent circadian effects. Furthermore, human-specific epigenetic markers such as DNA methylation in clock genes and transgenerational inheritance lack validation beyond preclinical F1-F2 data. In addition, most of the reviewed evidence characterizes static disease outcomes in offspring rather than their underlying physiological rhythmicity. Direct measures such as actigraphy-based sleep architecture, heart rate variability, and ambulatory blood pressure monitoring in offspring exposed to MCD remain scarce, particularly in human populations, representing an important direction for future research. Future research must focus on prospective human cohorts, the discovery of epigenetic biomarkers, and chronotherapy trials to enhance clinical translation.
